# Exploring the Goat Rumen Microbiome from Seven Days to Two Years

**DOI:** 10.1371/journal.pone.0154354

**Published:** 2016-05-02

**Authors:** Lizhi Wang, Qin Xu, Fanli Kong, Yindong Yang, De Wu, Sudhanshu Mishra, Ying Li

**Affiliations:** 1 Animal Nutrition Institute, Sichuan Agricultural University, Ya'an, Sichuan, China; 2 Institute of agriculture and technology, Panzhihua, Sichuan, China; 3 Farm Animal Genetic Resources Exploration and Innovation Key Laboratory of Sichuan Province, Sichuan Agricultural University, Chengdu, Sichuan, China; University of Queensland, AUSTRALIA

## Abstract

Rumen microbial communities play important roles in feed conversion and the physiological development of the ruminants. Despite its significance, little is known about the rumen microbial communities at different life stages after birth. In this study, we characterized the rumen bacterial and the archaeal communities in 11 different age groups (7, 15, 30, 60, 90, 120, 150, 180, 360, 540 and 720 days old) of a crossbred F1 goats (n = 5 for each group) by using an Illumina MiSeq platform targeting the V3-V4 region of the 16S rRNA gene. We found that the bacterial communities were mainly composed of Bacteroidetes, Firmicutes, and Proteobacteria across all age groups. The relative abundance of Firmicutes was stable across all age groups. While changes in relative abundance were observed in Bacteroidetes and Proteobacteria, these two phyla reached a stable stage after weaning (day 90). Euryarchaeota (82%) and Thaumarchaeota (15%) were the dominant phyla of Archaea. Crenarchaeota was also observed, although at a very low relative abundance (0.68% at most). A clear age-related pattern was observed in the diversity of bacterial community with 59 OTUs associated with age. In contrast, no age-related OTU was observed in archaea. In conclusion, our results suggested that from 7 days to 2 years, the ruminal microbial community of our experimental goats underwent significant changes in response to the shift in age and diet.

## Introduction

The rumen of ruminant animals is colonized by a complex microbiome consisting of bacteria, archaea, protozoa, and fungi. They play an important role in the host’s health and productivity [1]. The microorganisms in the rumen help degrade plant fibers into chemical compounds such as short-chain fatty acids and ammonia [2]. These compounds are subsequently absorbed and digested by the animals to meet the requirement for essential processes such as growth, thermoregulation, and immunity [3, 4]; they can also stimulate the development of the rumen wall villi [5, 6].

It was believed that the lack of sufficient understanding of the ruminal microbiome will hinder effective enhancement of rumen functions. Therefore, many studies have been conducted to investigate the structure and composition of ruminal microbiome since many decades ago, primarily using exclusively culture-based methods [7, 8, 9] and then using polymorphic analysis of 16S rRNA gene sequences [10, 11, 12]. Until now, plenty of information has been collected regarding the development of the ruminal microbial community. The rumen has been reported to be sterile at the moment of birth [13]. Whereas, in a recent study, methanogens and fibrolytic bacteria were found in the rumen of calves less than 20 minutes after birth [14]. The primary microbial community in the newborn rumen was mainly composed of aerobic and facultative anaerobic microbial taxa, which were replaced exclusively by anaerobic taxa at between 6 and 8 weeks of age [7, 8, 9]. Albeit the ruminal bacterial community composition showed distinct shifts between primiparous and multiparous dairy cows [15]. Thus, these studies indicated a change in structural and compositional properties of the ruminal microorganisms with age. However, most of the previous studies were focused on the changes occurring at the early stages of life after birth. There was only one report focused on the bacterial changes from birth to adulthood [11], where only 5 stages of age were investigated and some important life stages such as weaning were not included. Furthermore, only a little information about the developmental process of ruminal archaea with age was available. In view of this, the present study included 11 growth stages to investigate how the community composition of bacteria and archaea is altered in response to growing age of goat. This information will strengthen the understanding of the microbial ecology and their functional role in the rumen, and ultimately provide greater insights into modulating ration formulations to improve productivity.

## Materials and Methods

All animal handling and sampling work were approved by the Institutional Animal Care and Use Committee of the Sichuan Agricultural University under permit number DKY-B20130302. All experiments were performed in accordance with the approved guidelines and regulations.

### Animal handling and sample collection

The experimental animals used in the present study included 55 male F1 crossbred goats (purebred male Boer goat × purebred female Jianchang black goat). The dams were 3.14±0.42 (mean, SD) years old at lambing and were fed different diet during gestation (S1 Table) and lactation (S2 Table). The goats were divided into eleven age groups (7, 15, 30, 60, 90, 120, 150, 180, 360, 540 and 720 days, n = 5 for each group). These goats were randomly selected from specific age groups of a goat flock raised in the experimental station of the Panzhihua Institute of Agriculture and Technology, Sichuan, China. These goats were raised according to the common practices used for commercial goat herds in china: after birth, kids were housed together with their mothers in the same pen where they suckled their mother’s milk as sole food until day 15; between day 16 and day 90 kids were allowed to have free access to a starter feed (S3 Table) in addition to milk; at day 90 kids were weaned with the dams moved away to other pens; the kids still stayed at their original pens until 6 months old when they were moved to fattening pens; starting at day 90 the goats were offered a mixed ration (S4 Table) in equal amounts at 0800 and 1900 h each day with approximately 5% feed refusal till slaughter. The goats had free access to fresh water throughout the whole experiment.

A flexible PVC tube (2 mm of wall thickness × 6 mm of internal diameter) with about 160 holes of 2.5 mm diameter in the 15 cm-probe head (Anscitech Co. Ltd. Wuhan, China) was connected to an electric vacuum pump (7 mbar) and was inserted into the rumen of experimental goats via the esophagus to collect the rumen sample. About 25 mL of rumen fluid from each goat was collected approximately 2–3 h after the morning feeding as described in a previous study [10]. The first 5 mL rumen fluid was discarded to avoid any contamination with saliva and the remaining fluid was filtered through four layers of cheesecloth. The resultant ruminal liquid was stored at -80°C until DNA extraction. To collect the samples from different sites of the rumen, the tube was inserted into the rumen with different depth each time when each goat was sampled (e.g., the adult goats were approximately 110–150 cm, 180 days old kids were approximately 100–130 cm, 90 days old kids were approximately 70–100 cm, etc). All sampling processes were finished within a week.

### DNA extraction and 16S rRNA gene sequencing

Total genomic DNA was extracted from the rumen fluids using TIANamp DNA Isolation Kit (Tiangen Biotech, Beijing, China) according to the manufacturer’s protocol. The DNA was quantified on 2% agarose gel and by using nanodrop (Thermo Scientific). Archaeal and bacterial 16S rRNA genes V3-V4 region (from 349 to 806) were amplified from extracted DNA using barcoded primers Arch349F (5’-GYGCASCAGKCGMGAAW-3’) and Arch806R (5'-GGACTACVSGGGTATCTAAT-3') [16]. Each 50 μL PCR reaction contained 10 ng DNA, 39 μL Molecular Biology Grade water, 5 μL 10X Ex Taq Buffer with 5 μL dNTPs (2.5 mM each), 0.5 μL Forward Primer (50 pM), 0.5 μL Reverse Primer (50 pM), and 0.25 μL Ex Taq Polymerase (5U/μL) (Takara Bio Inc. Japan). PCR was performed under following conditions: 94°C for 3 min; followed by 30 cycles of 94°C for 30 s, 50°C for 30 s, and 72°C for 5 min; followed by a final extension at 72°C for 5 min. Three replicates of PCR were performed for each sample. These PCR products were pooled, purified and then quantified by using nanodrop (Thermo Scientific). Then, next-generation sequencing was performed by Illumina MiSeq 300PE which was conducted by Macrogen Inc. (Seoul, South Korea).

### Sequences analyses

Next-generation sequencing reads from different samples were identified by barcodes using QIIME V1.8.0 pipeline [17]. Low quality reads including those with uncertain nucleotides, continuous three nucleotides with Q value less than 20, and unmatched barcode sequences were removed in QIIME V1.8.0. Uchime algorithm [18] implemented in Mothur V.1.33.3 [19] was used to remove chimeric sequences. Sequencing noise was further reduced using a preclustering methodology [20]. Clean and high-quality sequences were then assigned to bacteria and archaea by the cluster command in Mothur. Downstream analysis for operational taxonomic units (OTUs) classification, alpha, and beta diversities were done separately for archaea and bacteria. A 97% similarity cutoff was used to define OTU by Mothur. To reduce biases caused by sequencing efforts, the number of reads per sample was randomly subsampled to 571 and 12821 for archaea and bacteria, respectively.

Good’s coverage, Alpha diversities including Inverse Simpson [21] and Shannon index [22], richness (observed number of OTUs) and evenness (Shannon evenness) were calculated using Mothur V.1.33.3 [19]. Beta diversities including the Jaccard index [23], Theta YC [24] and Bray-Curtis [25] were calculated to estimate the dissimilarities in community membership and structures. Principal coordinate analysis (PCoA) was applied to visualize the dissimilarity of microbial communities among different age groups. MaAsLin [26] was used to evaluate the association of gut microbial community with age of goat. Breed was not accounted in any statistical models since all animals have similar genetic background (F1 crossbred with 50% Boer and 50% Jianchang Black Goat). All sequence data in the present study have been deposited to the sequence read archive (SRA) of the NCBI database under number SRP068108.

## Results

### Composition of bacteria and archaea in different age groups

After quality control and chimera removal, in total 2,138,084 high-quality reads were retained. Among those, 1,696,969 with an average of 30,854 reads per sample were identified as bacteria. However, only 156,140 in total and an average of 2,839 reads per sample were identified as archaea. These sequences, with a median (interquartile range) length of 202 bp, were assigned to 4,356 and 79,728 OTUs of archaea and bacteria, respectively, based on 97% similarity cutoff. Here, only OTUs with at least two reads were counted. The average Good’s coverage was 95.83% ± 0.86% and 90.94% ± 1.48% (mean ± SD, S5 Table) for bacteria and archaea, respectively. It is noticeable that the abundance of bacterial community was much higher than that of the archaea (range from 0.8% to 27% in relative abundance) for all age groups, and the 7^th^-day rumens had the least proportion of archaea than other groups. However, the differences were not significant, except for the comparison between 7^th^ and the 180^th^, which was statistically significant (P < 0.05, two-tailed T-test). For the downstream alpha and beta diversity analysis, sequence number was normalized to 571 and 12821 for archaea and bacteria, respectively, by randomly subsampling to standardize sampling effort.

**Fig 1:**
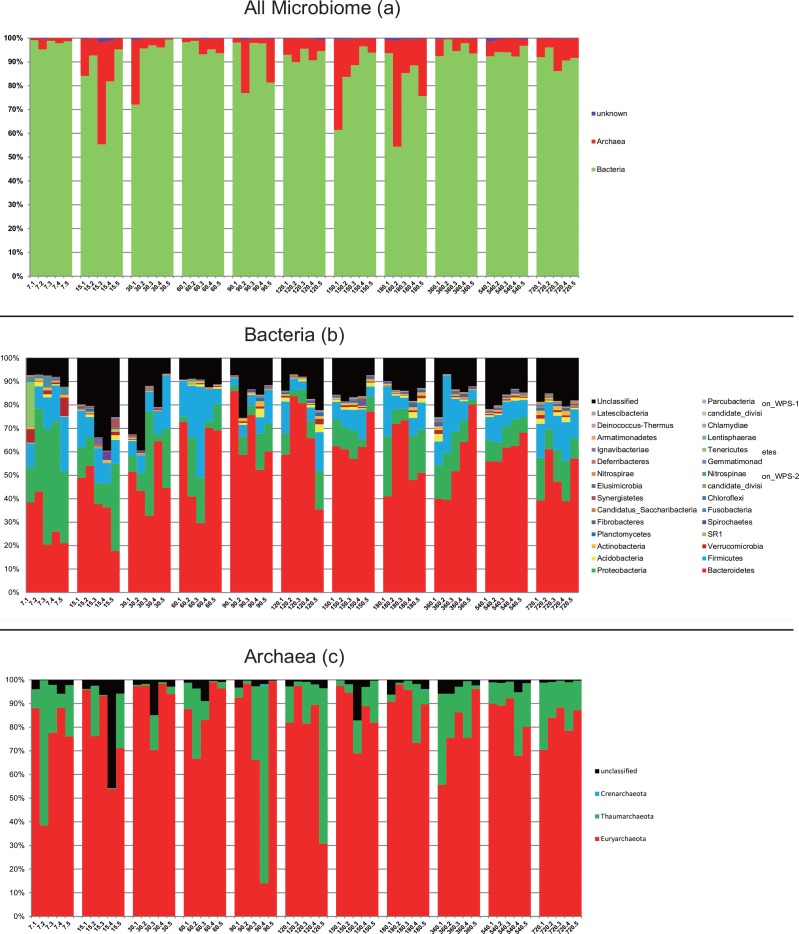
The compositions of all microbiome at Kingdom level (a), bacteria (b) and archaea (c) at Phylum level of goat’s rumen in different age groups.

**Fig 2:**
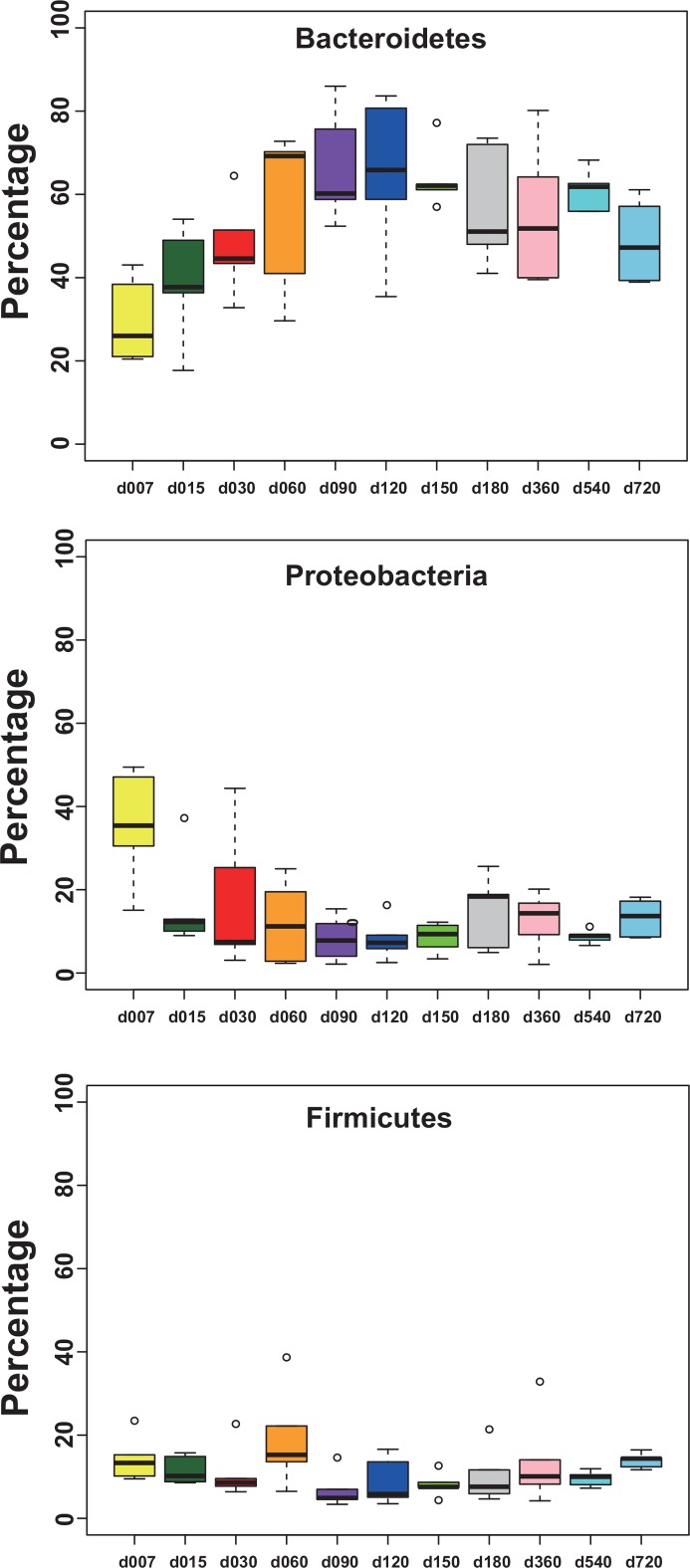
Relative abundance of the main Bacterial phyla: Bacteroidetes, Proteobacteria and Firmicutes. The top and bottom boundaries of each box indicate the 75th and 25th quartile values, respectively. The horizontal lines within each box represent the mean values.

Based on the taxonomic outlines of the SILVA project, 30 phyla were identified with Bacteroidetes (mean = 0.49, SD = 0.16 for all samples), Firmicutes (mean = 0.14, SD = 0.07) and Proteobacteria (mean = 0.13, SD = 0.11) as the dominant phyla (Fig 1). In case of Archaea, we found that Euryarchaeota and Thaumarchaeota were the dominant phyla which represented 82% and 15% of Archaea, respectively. We also found the presence of Crenarchaeota in some samples at a very low relative abundance (0.68% at most). Among different age groups, the abundance of Bacteroidetes increased from day 7 to day 90 and then became stable after that (Fig 2). This phylum at 7^th^-day of age was significantly lower in abundance than almost all other age groups except the 15 days age group (p < 0.05, two-tailed t-test). Whereas Proteobacteria decreased from day 7 to day 90, Firmicutes were relatively stable among different age groups (Fig 2). Relative abundance of archaea and bacteria at genus level was shown in S1 Fig.

### Rumen microbial diversities across age groups

**Fig 3:**
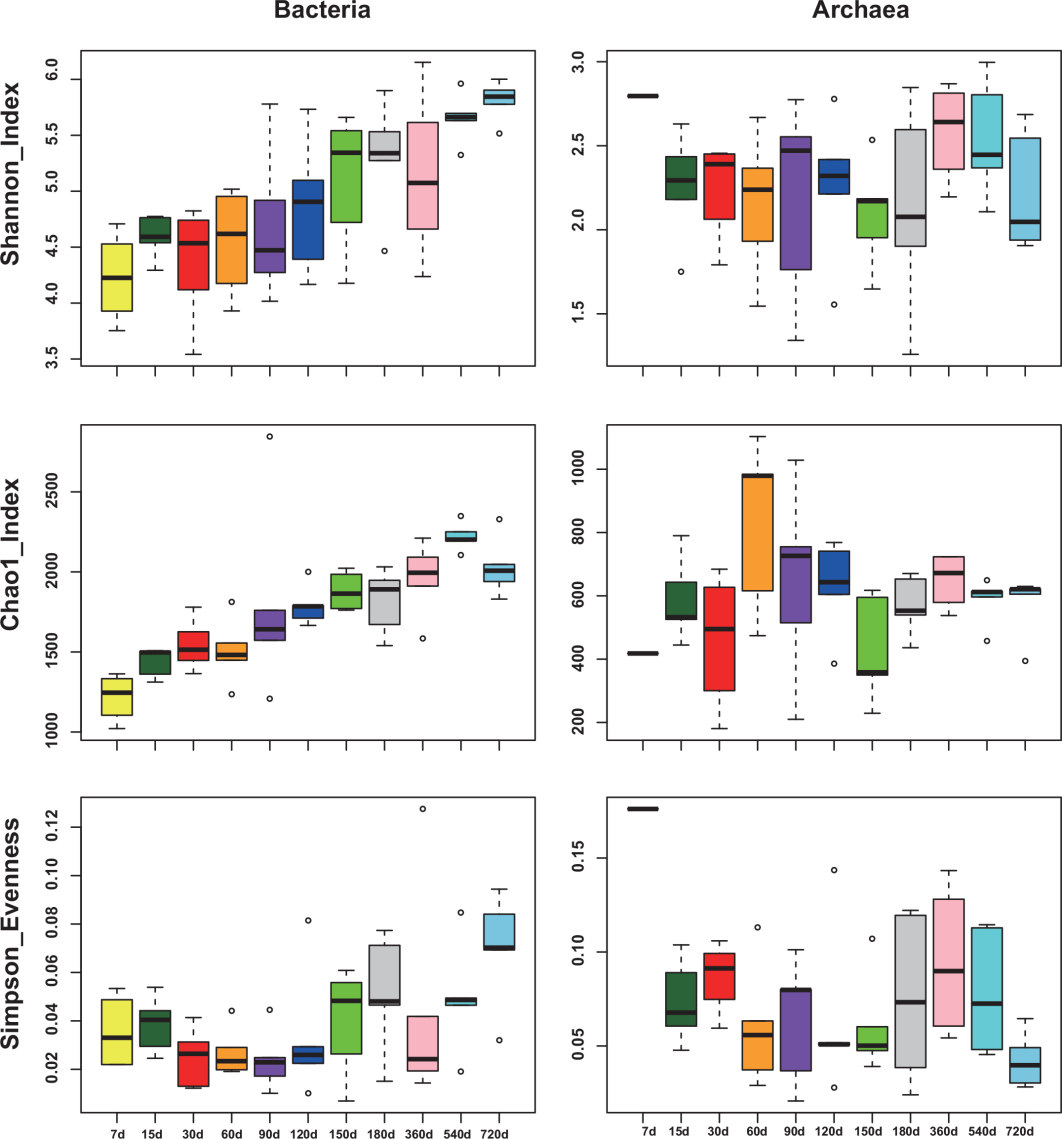
Alpha diversities within each age group in bacteria and Archaea respectively. Diversity was measured by Shannon index; Richness and evenness were measured by the Chao 1 and Simpson evenness index, respectively. The top and bottom boundaries of each box indicate the 75th and 25th quartile values, respectively. The horizontal lines within each box represent the median values.

Archaeal and Bacterial community diversity were measured by Shannon, Chao 1 and Simpson evenness (Fig 3) among different age groups. All three indices showed an increment in an age-dependent manner in rumen bacterial community. However, no similar pattern was observed in the archaeal community.

**Fig 4:**
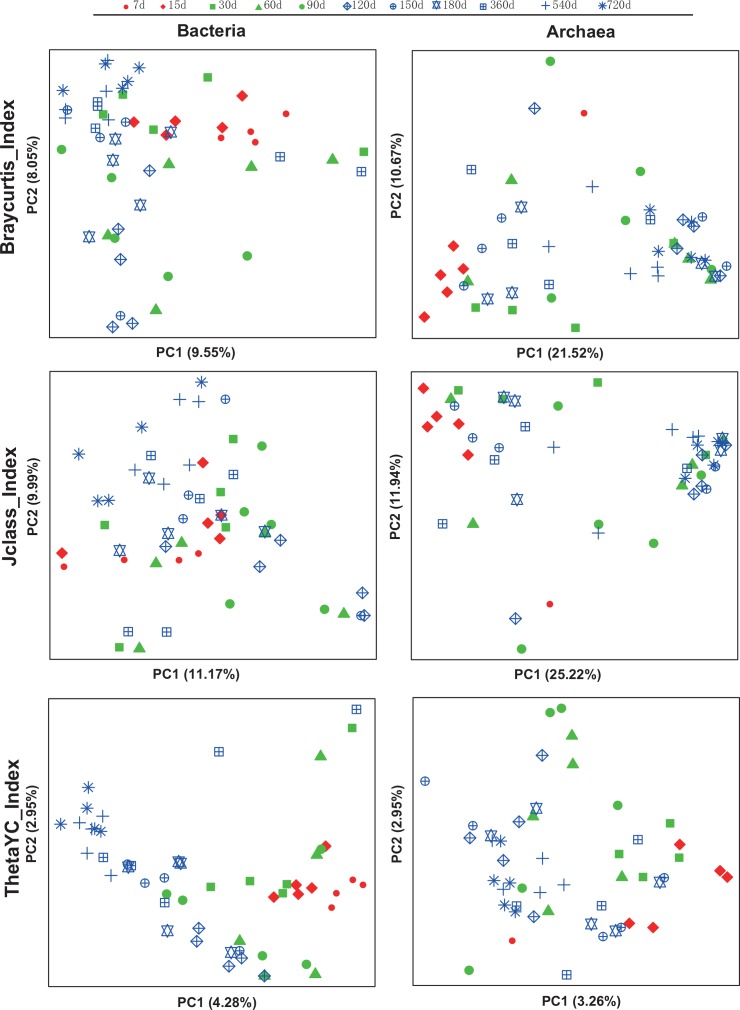
Principal coordinate analysis (PCoA) of the community structure using Bray-Curtis, Jclass and ThetaYC distances in Bacteria and Archaea of goats’ rumen. Different shapes represent different age groups and colors represent different diets.

We further calculated the Beta diversity, visualized by PcoA in Fig 4, with symbols representing different age groups and colors indicating different diets. No clear clustering patterns based on age groups or diets were observed in general. However, communities from 7 and 15 days old babies were more close to each other than to other communities, in both bacteria and archaea (Fig 4). A diet driven clustering pattern was observed in bacteria using Theta YC distance.

### Genus associated with age

We observed age-dependent increase in bacterial, but not archaeal, community diversity in the goat rumen. Next, we sorted to examine OTUs associated with age by using MaAsLin, a multivariate statistical method. The relative abundance of 59 OTUs (57 positives and 2 negatives) were associated with age (S6 Table) in bacteria. However, no OTU was observed in archaea to be associated with age.

## Discussion

In this study, Bacteroidetes, Proteobacteria, and Firmicutes were identified as the dominant phyla across all age groups in goats’ rumen. This was consistent with a report on cow [11] but is inconsistent with another study [12], in which the Synergistete was one of the dominant phyla representing over 30% of their total reads in goat rumen. This phylum only represents 0.13% on average in our data. Furthermore, previous observations found that the most abundant bacterial phylum in the rumen fluid of mature ruminants were Bacteroidetes or Firmicutes [11, 27, 28, 29]. However, a recent study reported that Proteobacteria accounted for 70% of the bacterial community in the ruminal fluid of calves on day 2 [30]. Jami et al. [11] also reported that the most abundant phylum in the rumen of 1 to 3 day old calves was Proteobacteria which represents 45% of the bacteria. They both noticed a sudden and sharp decline in the abundance of Proteobacteria after the neonatal stage. In the present study, we also observed changes in the abundance of Proteobacteria and Bacteroidetes with age. According to these trends, we divided the developmental process with age of the ruminal microbial community into two stages. The first stage could be defined between 7 to 90 days. During this stage, the abundance of Proteobacteria continually decreased, while Bacteroidetes continually increased with age. Rey et al. [30] studied the establishment of the ruminal bacterial community in dairy calves from birth to weaning and also found the abundance of Proteobacteria declined rapidly from 3 days of age, reaching the lowest values between 3 and 15 days while the phylum Bacteroidetes rose and became dominant. The second stage could be defined to after 90 days of age. The lowest abundance of Proteobacteria and the highest of Bacteroidetes all appeared at 90 days, after that, the abundance of Proteobacteria and Bacteroidetes remained relatively stable, so did the other bacterial taxa. However, the diversity of the community kept increasing until 360 days.

Many previous studies have verified the effect of dietary composition on ruminal community [31, 32, 33]. In the present study, the dietary composition also changed with age. Before 15 days of age, the goats only suckled their mother’s milk. Although most of the suckled milk went into abomasums directly due to the closure of the esophageal groove by reflex action [34], part of the milk leaked into the rumen [30] and became the only possible substrate for ruminal microbes. From 16 to 90 days, the kids had free access to solid starter except for suckling milk. During this period, with the growing age, the kids consumed more and more solid starter and least amount of milk, which led the substrate in the rumen increasing in fiber and decline in protein, starch, and fat. Therefore, it was reasonable to attribute the change of bacteria between 7 and 90 days to the combined effect of both age and diet. Early experiments also indicated that the separation of young animals from their mothers significantly induced psychological distress [35] and physiological stress [36, 37], which led to the shift and reestablishment of gastrointestinal microflora [38]. In the present experiment, before weaning, the kids lived together with their mother and their diet was composed of milk and starter, which was high in protein, starch, fat, and low in fiber (S3 Table). After weaning, the kids were separated from their mothers and were offered completely a mixed ration containing alfalfa meal and rice straw, which was high in structural carbohydrates (S4 Table). Therefore, the influence of weaning on ruminal microflora probably came from the combined effect of a shift in dietary composition, the stress of physiology and psychology, and change in age.

Typically, archaea populations contribute only 3–4% of the rumen microbiome, while Euryarchaeota was dominant archaea phylum in the rumen in previous studies [15, 39], which were similar to our findings too. In our experiment, we observed the existence of archaea in all the 7 days old kids, and all the archaeal genera existing in mature rumen were discovered in this group. Some early studies have reported that methanogens could be detected in rumen fluid of young kids of 2 days old [40,41]. In a recent study, Guzman et al. [14] reported the presence of the methanogens in the rumen of calves 20 minutes after birth. These findings suggested that the colonization of archaea in rumen began before solid carbohydrate presented in the rumen. The digestion of carbohydrate in rumen produces hydrogen as a byproduct, which was essential to the growth of methanogens. But, how the methanogens obtain hydrogen to maintain their growth and proliferation before solid carbohydrate ingestion remained unknown.

In this study, we found a clear age-dependent pattern in bacteria of goat rumen, indicated by bacterial composition (Fig 1), alpha diversity (Fig 2) and beta diversity (Fig 4). This is consistent with a previous study on rumen of cow [11]. However, similar age related pattern was not found in the archaea of goat’s rumen. Furthermore, no genus of archaea was identified to be associated with age, in contrast to 59 OTUs which were identified in bacteria. Comparing to other ruminal microbiome, archaeal communities seem to be less sensitive to the changes in age and diet [42, 43]. Kumar et al. [15] concluded that this might be due to either the low proportion of archaea (3–4% of total microbes) in the rumen or the establishment of stable archaeal communities in the rumen at a very early age. However, this result might also be due to the relatively small size for each age group of goats or the insufficient sequence depth. Future studies using larger samples or metagenomics (i.e. sequencing the whole rumen community at different age group instead of just the 16S rRNA gene) based approaches are required to address this question.

## Supporting Information

S1 Fig Relative abundance of Bacterial and Archaeal composition at genus level. Colors were assigned to genera with an average abundance > 0.5% for bacteria and 0.1% for archaea for all subjects.
(EPS)

S1 Table The ingredient composition and chemical contents of diets for pregnant goats. (DOCX)

S2 Table The ingredient composition and chemical contents of diets for lactating goats. (DOCX)

S3 Table The ingredient composition and chemical contents of starter feed for goats between 16 and 90 days old. (DOCX)

S4 Table The ingredient composition and chemical contents of starter feed for goats more than 90 days old. (DOCX)

S5 Table Numbers of sequences and Good’s coverage for archaea and bacteria. (XLSX)

S6 Table Multivariate Analysis to find associations between age and microbial community abundance at genus level for bacteria. No association between age and microbial community abundance were detected at genus level for archaea.
(XLSX)
