# Supplementary material for: Exploring the Goat Rumen Microbiome from Seven Days to Two Years
Source: PLoS One. 2016 May 2;11(5):e0154354. doi: 10.1371/journal.pone.0154354 (PMC4852915; doi:10.1371/journal.pone.0154354)
Supplement: S1 Table — (DOCX) [file pone.0154354.s002.docx]

**Table S1** The ingredient composition and chemical contents of diets for pregnant goats (%, DM basis)

| Ingredient composition |  | Chemical contents |  |
| --- | --- | --- | --- |
| Alfalfa meal | 30.7 | Crude Protein | 10.4 |
| Rice straw | 39.4 | Neutral Detergent Fiber | 53.8 |
| Corn | 9.8 | Acid Detergent Fiber | 32.4 |
| Wheat bran | 5.2 | Calcium | 0.91 |
| Corn bran | 2.2 | Phosphorus | 0.36 |
| Soybean meal | 1.8 |  |  |
| DDGS | 4.1 |  |  |
| Rapeseed meal | 5.0 |  |  |
| Dicalcium phosphate | 0.3 |  |  |
| Salt | 0.5 |  |  |
| Premix^a^ | 1.0 |  |  |
| Total | 100 |  |  |

^a^Premix provides: Ca (as calcium carbonate) 2.5g/kg DM; Fe(as ferrous sulfate) 30 mg/kg DM; Cu (as copper sulfate) 10 mg/kg DM; Zn (as zinc sulfate) 50 mg/kg DM; Mn (as manganese sulfate) 60 mg/kg DM; Vitamin A 2937IU; Vitamin D 343 IU; Vitamin E 30 IU.
