# Supplementary material for: Exploring the Goat Rumen Microbiome from Seven Days to Two Years
Source: PLoS One. 2016 May 2;11(5):e0154354. doi: 10.1371/journal.pone.0154354 (PMC4852915; doi:10.1371/journal.pone.0154354)
Supplement: S3 Table — (DOCX) [file pone.0154354.s004.docx]

**Table S3** The ingredient composition and chemical contents of starter feed for goats between 16 and 90 days old (%, DM basis)

| Ingredient composition |  | Chemical contents |  |
| --- | --- | --- | --- |
| Corn | 55.0 | Crude Protein | 16.8 |
| Puffing soybean meal | 15.0 | Neutral Detergent Fiber | 12.5 |
| Alfalfa meal | 12.0 | Acid Detergent Fiber | 3.7 |
| Wheat bran | 7.0 | Calcium | 1.10 |
| Whey powder | 8.0 | Phosphorus | 0.60 |
| Premix^a^ | 0.5 |  |  |
| Salt | 0.5 |  |  |
| Calcium carbonate | 1.3 |  |  |
| Dicalcium phosphate | 0.7 |  |  |
| Total | 100 |  |  |

^a^Premix provides: Fe(as ferrous sulfate) 55 mg/kg DM; Cu (as copper sulfate) 20 mg/kg DM; Zn (as zinc sulfate) 80 mg/kg DM; Mn (as manganese sulfate) 100 mg/kg DM; Vitamin A 5630IU; Vitamin D 672 IU; Vitamin E 58 IU.
